# The Case of the Late Emperor Frederick

**Published:** 1889-02

**Authors:** 


BUFFALO MEDICAL AND SURGICAL JOURNAL

A MONTHLY REVIEW OF MEDICINE AND SURGERY.

EDITORS:

THOS. LOTHROP, M. D., - - - W. W. POTTER, M. D.

All communications, whether of a literary or business character, should be addressed to the editors: 284 FRANKLIN STREET, BUFFALO, N. Y.

### Editorial.

### *THE CASE OF THE LATE EMPEROR FREDERICK.*

Except in a very brief and general way, this journal has refrained from discussing the different points at issue between the German and English physicians, who were engaged in the treatment of the late Emperor Frederick; and it would not attempt any discussion of these points were it not for the fact that the spirit of unfairness and unjust criticism has seemed to so influence the mind of many who have assayed to do so.

It is profoundly to be regretted by the whole medical world that this controversy has arisen in the medical ranks, and that all the strife and bickerings that were indulged in by some of those in attendance upon him during his illness, did not cease with the going out of his noble life. Were the medical world to derive any special benefit, or were any light to be thrown upon any obscure scientific pathological points by this controversy, there might have been a good excuse for its continuance. But, as a matter of fact, it has consisted mainly in personalities and in giving vent to petty jealousies, by bringing forward serious charges under the shadow of a scientific cloak.

In discussing the controversy as it now stands, we will first take a general glance over the report put forth by the German physicians. This report professes to be "an authentic record of the illness of the Emperor Frederick III., derived from official sources and founded upon the reports deposited in the archives of the royal house of Prussia." The report consists in individual reports of ten ot the eighteen German physicians, who were at one time or another engaged in the case.

It is a relevant question to ask in this connection why it is that the editor, Prof. von Bergmann, selected but ten of the eighteen German doctors who were connected with the case, to make up this report? He has evidently selected these reporters, as certain writers have pertinently suggested, with "professional prudence," for the opinions of the remainder were not in accordance with Prof. von Bergmann's view of the case.

We would naturally look for a report from Dr. Wegner, the Crown Prince's personal physician, and who had watched the case from the beginning to the end. We also find no report from Virchow, who would be the most important witness against Sir Morell Mackenzie. We have none from Krause, one of the leading and most able of Berlin throat specialists, and who had much to do with the case. We also have no report from Hahn, who was connected with the early history of the case, and who stated that without pathological evidence of a malignant disease in the larynx, he could not recommend external operations. Von Lauer and Schræder, both eminent laryngologists, and who were present at the consultation in June, 1887, when it was decided to postpone the operation, are also silent.

Sir Morell Mackenzie's reply is divided into three parts, historical, controversial and statistical. In the historical portion he gives a complete and consecutive report of the case from the time that he was summoned to Berlin, May 18, 1887, until the death of the Emperor, on June 15, 1888. This portion of the report contains twenty-one illustrations, showing the condition of the late Emperor's larynx at different periods; the various cannulæ used after the operation of tracheotomy; the manner in which Mr. Howell made his measurements in proving the asymmethical position of the tracheal incision, and the false passage made by Prof. von Bergmann in his attempts to push a cannula into the windpipe. The clinical progress of the case during this period is very minutely and concisely detailed, the minutia of which must be, more of less, familiar to our readers.

In the controversial portion, Sir Morell not only denies the specific charges made by the German physicians, but asserts that by unskilful and bungling management of the case, the life of the patient was very materially shortened, and shows that the rosy hope held out at the beginning of the case by the performance of what they termed a trivial operation, was not only illusory but unwarrantable, without positive evidence of a malignant disease.

The statistical portion of the book gives a tabulated report and analysis of all cases of thyrotomy, of partial extirpation of the larynx, and of total extirpation of the larynx.

The first and main charge made by the German doctors is that Sir Morell Mackenzie was entirely responsible for the postponement of the operation in May, 1887, and thereby sacrificed the life of the Emperor.

At the time of the consultation of the three German physicians, they stated that they were, at that time, absolutely positive that the disease affecting the Crown Price was cancer, and had determined upon the operation of thyrotomy or laryngofissur for its extirpation. Their desire, however, to call in the authority of a specialist, the most eminent that could be found, was an acknowledgment, on their part, of some doubt existing in their minds as to the correctness of their diagnosis. Sir Morell Mackenzie was consequently called from London, not, however it would seem, because they expected his opinion to be of any value, but because, as Prof. Gerhardt states, "every per son who knows how to make a laryngoscopic examination must come to the same conclusion." Sir Morell Mackenzie did not concur in their opinion, but believed that the appearance of the growth in the larynx was not sufficiently characteristic of malignant disease to warrant so serious an operation as that of thyrotomy, for which the Germans had prepared themselves to perform, and he did not think the operation justifiable without pathological evidence of the disease. Sir Morell then, at the request of the German physicians, proceeded to remove a portion of the growth, which was submitted to Virchow for microscopic examination, who said that the portion submitted to him was very much diseased, but not of such a character as to excite apprehension. With this pathological evidence that it was not cancer, made by the most skilful pathologist in the world, it would have been foolhardy to have undertaken the operation proposed.

One of our leading American journals, in discussing this point, very truly says :

"In the light of American conservative surgery, we do endorse Mackenzie's procedure in first seeking Virchow's report as to the microscopic appearance of the growth, before accepting the theory of cancer, especially when that acceptance was the first step to an operation of the gravest and most doubtful import. Virchow's report justified Mackenzie's position, and after the reception of the report, which was negative in its conclusions as to cancerous conditions, even they who advocated laryngeal excision could not consistently demand it in the face of Virchow's great pathological authority. To illustrate : Suppose excision had been performed at this time, and that the patient had succumbed, either under the knife or a few days or weeks afterwards, as is commonly the case—suppose, then, the report of Virchow upon the excised portion had been the same as he did give, showing no cancerous condition present. What would then have been the professional and lay verdict against the physicians responsible for the seemingly needless and hazardous performance ? Unexcusable and unpardonable."

The German physicians, therefore, at once concurred in postponing the radical operations until pathological evidence warranted its performance. Hence, the German physicians were equally responsible with Mackenzie, for had they not approved of this plan, they should, in justice to themselves, have withdrawn from the case, as Sir Morell in his answer points out.

Sir Morell Mackenzie in his reply also shows, by the statistics which Prof. von Bergmann has incorrectly quoted, that the operation of thyrotomy (laryngofissur) is no trivial affair. To prove the harmlessness of the operation, Prof. von Bergmann states that there appeared in the *Centralblatt für Laryngologie*, since its publication four years ago, reports of fifteen cases of thyrotomy (laryngofissur), only one of which proved fatal, in which instance death was due to diphtheria. This statement Sir

Morell Mackenzie shows to be misleading, for the operation was actually reported in the *Centralblatt* no less than thirty times; but the disease was cancer in only ten cases. Of these ten cases only five patients survived the immediate effects of the operation, and only one patient remained free from the disease for over two years.

With our present antiseptic methods, which have so much increased the safety of all operative procedures, the operation of simply opening the larynx on the median line would seem to be a less grave operation than the statistics show it to be. The risk in the operation, however, does not consist entirely in the simple opening of the larynx, but in the complications that may arise, such as inflammation of the lungs and air passages, which so frequently follow these operations, and from which the patients rarely recover, and which the most rigid carrying out of the antiseptic method has little or nothing to do in preventing. The difficulty of entirely removing a cancerous growth from the interior of the larynx, by scraping it out after the preliminary operation of thyrotomy, and the al rming hemorrhage which so often occurs, is pointed out by all writers on the subject who have had experience in performing this operation: such as Prof. Navretil, Prof. von Schrötter, Mr. Timothy Holmes, Mr. Davies-Colley, and Dr. Fauvel. Dr. Gottsstein, of Breslau, says in his very able work on diseases of the larynx, that the extirpation of malignant neoplasms by thyrotomy, has in his personal experience given very unsatisfactory results. We have, therefore, every reason to believe that had opening the larynx and scraping out the interior been performed, and the patient survived the immediate effects of the operation, if the disease was at that time cancer it would have returned in a short time; then the patient would have been in a far worse condition than before, and the disease would not have pursued a comparatively painless course as it did under the treatment adopted by Dr. Mackenzie.

The charge made by Prof. Gerhardt that Sir Morell Mackenzie, in attempting the removal of the laryngeal growth, had wounded the opposite vocal cord, was simply absurd in the light of Sir Morell's marvelous skill. The condition of the patient afterward shows that no serious injury could have been done; and even were it so, the manner in which this charge was made at the time by Prof. Gerhardt, openly before the patient, was most shockingly unprofessional.

The charge is also made by Prof. Gerhardt, that Sir Morell Mackenzie, in operating, took the forceps out of his pocket and introduced them into the patient's throat without first disinfecting them, by which he attempts to show that Sir Morell did his work in a very careless manner. This is simply disposed of by the statement of Sir Morell himself, that the instruments were carried in a silk bag, lined with carbolized wool, showing that the instruments were already in a condition of the utmost cleanliness.

Prof. Gerhardt also charges that, in operating, the light fell on the patient's cheek instead of on the laryngeal mirror; to which Sir Morell replies, that in reflecting a ray of light on a mirror it must pass before the face of the patient before it reaches the little glass; if, therefore, Prof. Gerhardt happened to notice it in its transit, it did not certainly warrant the charge that the light did not reach the laryngeal mirror. No one would attempt to introduce the laryngeal forceps without first illuminating the larynx.

Another charge brought by Prof. Gerhardt in his report is, that Sir Morell Mackenzie during the consultation in Berlin, in May, had promised the relatives of the illustrious patient to cure the disease in a few weeks. Any one acquainted with the treatment of growths in the larynx, would know that this accusation could not be true, for the cure of even benign growths in the larynx is most uncertain; and Sir Morell Mackenzie states in his reply that the most he ever said on the subject was that "if the disease is not cancer, I believe I shall be able to cure it."

He is also accused of recommending the Isle of Wight as a climate that would greatly assist in the cure. It was arranged, previous to the time that this operation was proposed, that the Crown Prince should attend the Peace Jubilee in London. As he was quite able to travel, he decided to go there, where he could, at the same time, be under the care of Sir Morell Mackenzie. The Isle of Wight was simply chosen as the place where he could remain the most quiet, and be free from the necessity of using his voice and seeing his friends.

Another principal charge brought against Sir Morell Mackenzie in which he is held responsible for the life of the Emperor, is that he delayed the operation by not arriving at the conclusion that the disease was of a cancerous nature, until it was too late to perform complete extirpation of the larynx. Those who make this charge seem to assume that neither the wishes of the patient, nor the desires of his family, were to be considered any more than are those of the patients in a general hospital.

It will be remembered that in November, after the Crown Prince had gone to San Remo, Italy, the disease took on an active form, and it was then unanimously decided by the physicians that the disease was of a malignant nature. At that time the two courses to be pursued, extirpation of the larynx or that of tracheotomy, were plainly laid before the patient, and he at once replied that "he could not consent to the major operation, but could only agree to tracheotomy when it became necessary;" and at no time during the course of his disease had he consented to the operation of extirpation of the larynx.

At this period of the disease, *The British Medical Journal*, of Nov. 19th, after briefly referring to the course which Sir Morell Mackenzie pursued in the case, says: "It is a fact that so lately as three weeks ago Dr. von Bergmann said to a high official on the staff of the Crown Prince, that he considered the course which Sir Morell Mackenzie recommended and pursued in the spring had been the right one. Under the circumstances it ill becomes any one now, wise as he may imagine himself, with the light thrown by a further development of events, to say that the course originally pursued was not the correct one."

Sir Morell in his reply, in reviewing the charges brought by his German accusers, also shows that they are open to criticism—in some instances of a serious nature—and most severely criticises the unwarrantable treatment that the Emperor received at the hands of Prof. Gerhardt at the commencement of the disease, by the repeated daily cauterizations of the larynx with the electric cautery. This cauterization might not have been the means of transforming a benign growth into a malignant one, but we think that the early appearance of perichondritis must have been due to this cause; for, if Prof. Gerhardt did not possess the manipulative skill to remove the growth with the forceps, he certainly could not have possessed the skill to have confined his cauterizations entirely to this spot; as this requires still greater dexterity than simply removing the growth.

Sir Morell also points out that the incision in the trachea made by Dr. Bramann, was three millimetres from the median line, as Mr. Hovell demonstrates by his ingenious measurements. As no serious ill effects resulted from this mishap, Dr. Bramann should not be unjustly criticised; but the persistent employment of ill-fitting tracheotomy tubes, which so greatly irritate the trachea, should not be overlooked.

The most serious charge is brought by Sir Morell against Prof. von Bergmann, when on April 12th, owing to some trouble with the tube, Sir Morell very courteously sent for the German Professor to consider the case with him. The details of this interview have since been very frequently referred to with reference to the conduct of Dr. von Bergmann, who arrived in a very excited manner, and very unceremoniously forced an unguarded cannula down into the tissues of the Emperor's neck; and, after several unsuccessful attempts at finding the tracheal opening, he thrust his finger down into it, when he was obliged to call his assistant to introduce the cannula for him. The false passage thus created resulted in an abscess, which Sir Morell unhesitatingly claims very materially shortened the Emperor's life. The production of a false passage was denied by Prof. von Bergmann, and at the autopsy its existence, it is claimed, was not demonstrated; but the official reports immediately following this occurrence describe an abscess that had formed in the patient's neck, and the pouring out through and around the tube of quantities of pus—a fact which was then inexplicable to us, and not in accordance with the clinical history of such cases.

The explanation why this abscess cavity was not distinctly observable at the autopsy, has, we believe, been amply explained by the fact that, at the time of the Emperor's death, the walls of this abscess had become so broken down and the tissues so much disintegrated, that the abscess had emerged into the one large cavity surrounding the tube.

In reviewing this controversy, there is one main thing to be borne in mind, namely: that the German physicians are the attacking parties, and that the reply of Sir Morell Mackenzie has been made purely in self-defense. The Germans claim that their statements were called forth, in order to place themselves correctly on record in reply to criticisms that have been made by the secular press. On this point, however, Sir Morell had quite as much cause of grievance as the German physicians, as is evident from the fact that the hostility against him extended to threats of personal violence, and even to the taking of his life. The attack of the German physicians was not only uncalled for, but positively without excuse. Notwithstanding their grievances from the criticisms of the secular press, to which they should have paid no attention, a recent writer very truly says: "The pride of their professional reputation, pride in the honor of the German science, alike dictated silence," but "in the bitterness of their mortification the German doctors forget the honor of their profession, their personal pride, the dignity of German science, their own self respect, and the teaching of experience."

Under these circumstances it is quite incomprehensible why Sir Morell Mackenzie should be criticised for replying in his own defense. To deny him this, is to deny him that which is provided for the most despised criminal at the bar of justice. It was only the multiplicity of the charges against his honor and against his skill, that provoked him to retaliate in the same manner in which he had been attacked.

The recent action of the English doctors in censuring Sir Morell for the publication of his book, and in charging him with divulging professional secrets, should not escape comment. It is a well-known fact that there has existed in London, for a long time, a spirit of rivalry akin to jealousy, growing out of his marvelous professional success, and this opportunity has been embraced to attempt, under the guise of professional ethics, to degrade Sir Morell.

On October 13, 1888, there appeared in the *British Medical Journal*, a paragraph giving evidence of the manner in which Prof. von Bergmann treated the late Emperor at the time when the alleged false passage was made. In this paragraph a facsimile is given of a portion of one of the Emperor's written communications. In this he remarks: "The same Hovell just tried before Bergmann illtreated me." This passage the editor positively states was obtained from outside sources, and not from those who had anything to do with the case. In the memorial which was drawn up by the English physicians, the signers protest against the appearance of this communication in the *British Medical Journal*, as a violation of professional confidence.

Notwithstanding the fact that the editor avowed that Sir Morell had nothing to do with it, some medical journals, and some of the profession as well, have been quick to use this circumstance as an occasion for expressing their disapproval of Sir Morell's professional conduct. Those who are so quick to seek an opportunity to find fault with Sir Morell, seem to overlook the ethical shortcomings of his accusers. They forget the fact that the first intimation that the Crown Prince received that he was suffering from a malignant disease, was through the reports given by Prof. Gerhardt to the outside world. A more shocking illustration of the portrayal of professional secrets, or the open disclosure of supposed facts obtained in a professional capacity, cannot be imagined, than the circumstance that Dr. Moritz Schmidt proclaimed to the world, and also made it the subject of a clinical lecture, that the Crown Prince was suffering from a specific disease. Notwithstanding the fact that Prof. Schrötter characterized this as "an old wife's tale," it would be in our courts a charge answerable to the law for defamation of character; and yet Sir Morell Mackenzie's English friends entirely overlook this, nothwithstanding the fact that ethics are supposed to deal with subjects too fine for the law to reach. We do not wish to set ourselves up as arbitrators between the German and British doctors, but we do dislike to see injustice done to one for the purpose of exonerating another.

The ethical point in this case which should not be overlooked, is the question of responsibility. The special charge in the German manifesto against Sir Morell is, that they held him entirely responsible for the termination of the case, because they consented to follow his advice. Suppose, for argument's sake, that we grant this point; the responsibility is then no more thrown on Sir Morell than before. When the German doctors accepted the plan proposed by Sir Morell, they became a party to that plan as much as if they had proposed it themselves. It is an unwritten law underlying ethics, if one seeks advice from another, the one who accepts advice and follows it is equally responsible; but in law, the one who accepts this advice and follows it, becomes entirely responsible for his own acts. In the case of a disastrous result from the following of advice sought for, the acceptor of the advice cannot say that the giver was foolish in bestowing such advice, and that he is, therefore, exempt from being considered foolish in following the same.

In all medical consultations, if the advice of the consultant is accepted by the attendant, they both become equally responsible, and in every instance, in so far as the one subscribes to the advice given by the other, they both share in the responsibility equally; and from this there is no escape.

There is also the serious charge of manifest unfairness by which the German physicians hold themselves open to adverse criticism. Their report, containing the most serious charges against Sir Morell, is permitted to circulate freely throughout the German empire, while Sir Morell's reply is, through their influence with the governmental authorities, at once suppressed and confiscated. This very clearly shows that there is a political motive underlying the whole affair, particularly when we see the young Emperor allowing himself to become so far influenced by this faction, as to become so unnatural a son as to openly denounce his own mother, and when, at the same time, this "hated Englishwoman" is none other than the daughter of an honored German prince.

We have spoken freely and fully, because of the great interest in this case, both professional and lay; and because, further, we dislike to see American politics influencing, as we fear has been the case, a calm judgment upon its scientific aspects, without doing whatsoever lies in our power to correct an erroneous medical opinion made up in such false light.

We have waited until the evidence was in, and now submit the foregoing to our readers as the most impartial analysis we have been able to make of it.

